# Use of video-electroencephalography as a first-line examination in veterinary neurology: development and standardization of electroencephalography in unsedated dogs and cats

**DOI:** 10.3389/fvets.2024.1326165

**Published:** 2024-01-26

**Authors:** Elsa Lyon, Hervé Pochat, Stéphane Blot, Thibaut Troupel, Nicolas Van Caenegem, Stéphane Besnard, Catherine Escriou

**Affiliations:** ^1^Laboratory Vertex, UFR STAPS Department, UniCaen, Caen, France; ^2^Small Animal Internal Medicine Department, Neurology Service, VetAgro Sup, Marcy l'Etoile, France; ^3^Amset Medical, Saint-Maur-des-Fossés, France; ^4^U955-IMRB, INSERM, Small Animal Internal Medicine Department, Neurology Service, Ecole Nationale Vétérinaire d'Alfort, Maisons-Alfort, France; ^5^Neurology Service, AniCura CHV Pommery, Reims, France; ^6^Neurology Service, AniCura TRIOVet, Rennes, France

**Keywords:** EEG, video-EEG, gel electrode, canine, feline, epilepsy, encephalopathy

## Abstract

**Objective:**

To assess the feasibility and validate the use of video-electroencephalography (EEG) in conscious dogs and cats and to propose guidelines of routine EEG in veterinary clinical practice.

**Design:**

Prospective clinical study.

**Data:**

One hundred and fifty EEG recordings were carried out to validate the clinical adding-value, reproducibility, and guidelines on 140 owned animals. One hundred and one EEGs were performed on dogs and 49 on cats.

**Procedures:**

We compared recordings performed with 8 EEG unwired stud Ag/AgCl electrodes held by elastic straps and 8 EEG wired cup Ag electrodes held by a tailor-made manufactured headset combined with a wired video-EEG device. Electrodes placement was determined according to previously published animal EEG protocols. Physiological sensors, such as electrocardiography, electromyography, and respiratory sensors were added. Stimulation protocols were tested. Quality and interpretability were evaluated.

**Results:**

Headsets and recording procedures appeared suitable for all skull shapes and sizes. Video-EEG recordings were successfully performed without tranquilization or anesthesia except for 9 animals. Median EEG recordings time was 40 min. Impedance remained below 20 kΩ in 99% of dog EEGs and 98% of cat EEGs. Isosynchrony was reported in 6% of the channels. Seventy-five percent of dog EEGs and 83% of cat EEGs were readable for more than 50% (to 100%) of their duration. Successful discrimination of vigilance states from rhythm analysis (wakefulness, drowsiness, and sleepiness) was possible in 99% of dog EEGs and 91% of cat EEGs. Photic driving responses during photic stimulations were observed in 11% of dog EEGs and 85% of cat EEGs. Electroencephalography recordings were directly informative in 32% of the examinations: in 25% EEG abnormalities were associated with clinical signs and 7% concerned EEG abnormalities without clinical symptoms during recording. Thirteen percent of dogs subjected to photic stimulation exhibited epileptic anomalies. Among 9 EEGs with other history-based stimulations, three displayed epileptic graphoelements.

**Conclusions:**

We have developed a standardized unanesthetized video-EEG procedure easily performed and reproducible in dogs and cats. Qualitative and quantitative technical and medical criteria were evaluated and were in accordance with human EEG recommendations. Moreover, we have demonstrated its relevance and accuracy for diagnostic purposes, providing further arguments for the use of EEG as a first-line neurological functional exploration test.

## 1 Introduction

In human medicine, electroencephalography (EEG) and video-EEG are widely and routinely implemented in various fields, including neurology, intensive care (for coma evaluation), neuropediatric, gerontology, and emergency medicine. In particular, EEG is used in epileptology, and its use allows for a finer classification of epilepsies, extending beyond determining seizure types and epilepsy categories to include electro-clinical characterization and the description of epileptic syndromes (1). The EEG recording procedures have been standardized from international and national recommendations, covering aspects such as the numbers and placement of electrodes, duration of examination, recording parameters and settings, choice of stimulation protocols and video analysis for seizure investigations in accordance with the medical context (2–6).

In veterinary neurology, EEG is used confidentially while epilepsy is one of the most common neurological disorder in dogs (7, 8) with an estimated prevalence ranging from 0.6 to 0.75% (8, 9). However, the prevalence of this disease is significantly higher in specific breeds, with reported prevalences of 3.1–33% (10–16) with varying clinical presentation and disease severity (10). Seizures are also common in cats and may account for 2% of reasons for veterinary visits (17) with recurrent seizures representing an estimated prevalence of 0.16% (18). However, feline epilepsy remains poorly characterized and epilepsy of unknown cause is reported in 22% of cats with seizures (19).

Current classifications of seizures and epilepsy in dogs and cats are only based on seizure semiology and epilepsy etiology, respectively (20). Epilepsy includes idiopathic epilepsy with a proven or suspected genetic background or an unknown cause and no indication of structural epilepsy and structural epilepsy, caused by identified cerebral pathology (20). The difficulty to perform easily EEG in veterinary practice is probably the main factor underlying the difference between human and companion animal epilepsy classification. The development of veterinary EEG could significantly enhance the diagnosis, classification and treatment of companion animal epilepsy (21). Recently, there has been a renewed interest in veterinary medicine, with the publications of protocols providing information on electrode positioning on the skull (22–25), suggested electrode types (23), anesthetic protocols (22, 26, 27), the feasibility of recording on animals without anesthesia using video-EEG (24) and interpretation (28). A survey on veterinary neurologists' EEG practices (29) reveals the variability in protocols employed regarding the use of video, recording durations, and assessment of recording quality through impedance measurements. This survey specifically shows a preference for subcutaneous, wired or needle electrodes over surface electrodes. Placement methods may adhere to published protocols (22, 24, 30, 30–34) or individual approaches often involving varying sedation protocols. Additionally, the survey highlights that EEG isn't routinely used by veterinary neurologists, with some performing it less than once a year. Challenges cited include limited access to EEG equipment and insufficient training and experience in conducting and interpreting EEGs.

Our aim was to develop a method and standards for routine EEG examinations in veterinary medicine similar to those used in human patients in unsedated conditions. This involved using cup electrodes or electrode caps and recording sessions lasting 20–30 min during medical visit as mentioned in recommendations (3–6). From a cohort of 230 dogs and cats, in various physiological and pathological contexts, we selected and tested electrodes and positions, developed and validated a method of recording under vigil conditions, without pain, restrain and learning, leading to recommendations and better practices for routine EEG investigations in veterinary medicine. In this article, we present the full methodological section and its validation including the detailed procedure, the evaluation of recording quality, artifact discriminations and illustrations showcasing physiological and pathological EEG patterns.

## 2 Materials and methods

The Ethics Committee of VetAgro Sup (No. 18) issued a favorable opinion (No. 1966) on the experimental protocol on November 28, 2019.

### 2.1 General procedures

Electroencephalography recordings were performed in cats and dogs presented to neurology unit at the veterinary campus of VetAgro Sup and École nationale vétérinaire d'Alfort (ENVA). Included animals were either brought by their owners for medical consultation or hospitalized for a short period due to brain disorders. Unconscious animals (i.e., animals with coma and status epilepticus) were excluded from this study. History, clinical signs, and results of diagnostic investigations were collected. We intentionally avoided using restrictive criteria in this study to demonstrate the method's reproducibility for dogs and cats across various medical contexts and to prevent selection bias.

The recordings were conducted after the consultation, either immediately or by appointment in following days. They were carried out by one of the authors (EL) in the presence of at least the animal's companion (owner or clinician), except for two examinations for which the operator was alone.

The examination rooms were customized to minimize visual, auditory, and olfactory stimulation and to be comfortable. They were clean, equipped with an examination table and a table for placing the acquisition device, chairs, sleeping mats and treats for dogs and cats. It was possible to create darkness in the room for photic stimulation, and a night light provided sufficient illumination light for recording.

Owners were advised to feed and take their animals out before the examination. They could bring their pet's sleeping mate and favorite treats. The recordings were mostly often performed with the dogs lying on the floor, with or without a mat, and the cats in their transport box, often with the top open, and placed on the examination table. Recordings were also operated with the animal on the owner's lap. In case of excessive heat during summer, a refrigerated mat was provided to the animal to prevent polypnea.

### 2.2 Device

EEG recordings were made using a wired EEG device (Brainbox^®^ 1042 Braintronics BV, Fl. The Netherlands) with EEG software (Coherence^®^ 7.1.3.2037 Natus Europe GMBH, Planegg, Germany). The acquisition settings were sampling frequency per channel 256 Hz, high pass filter 0.3 s, low pass filter 35 Hz, resolution 7 μV/mm, longitudinal and transverse montages. These montages were preferred over the referential montage to avoid contamination of the reference electrode by artifacts, especially cardiac, and to maximize the chances of observing small focal potentials (30, 31). A 50 Hz filter was used to avoid disturbances related to alternating current. These settings are those recommended in human medicine, except for the low-pass filter, which was reduced from 70 to 35 Hz in order to limit muscular artifacts without restricting brain rhythms observation (2–6). This device allowed synchronized video and EEG recording, as well as the configuration and visualization of light protocols.

### 2.3 Electrodes and cap

**Figure 1:**
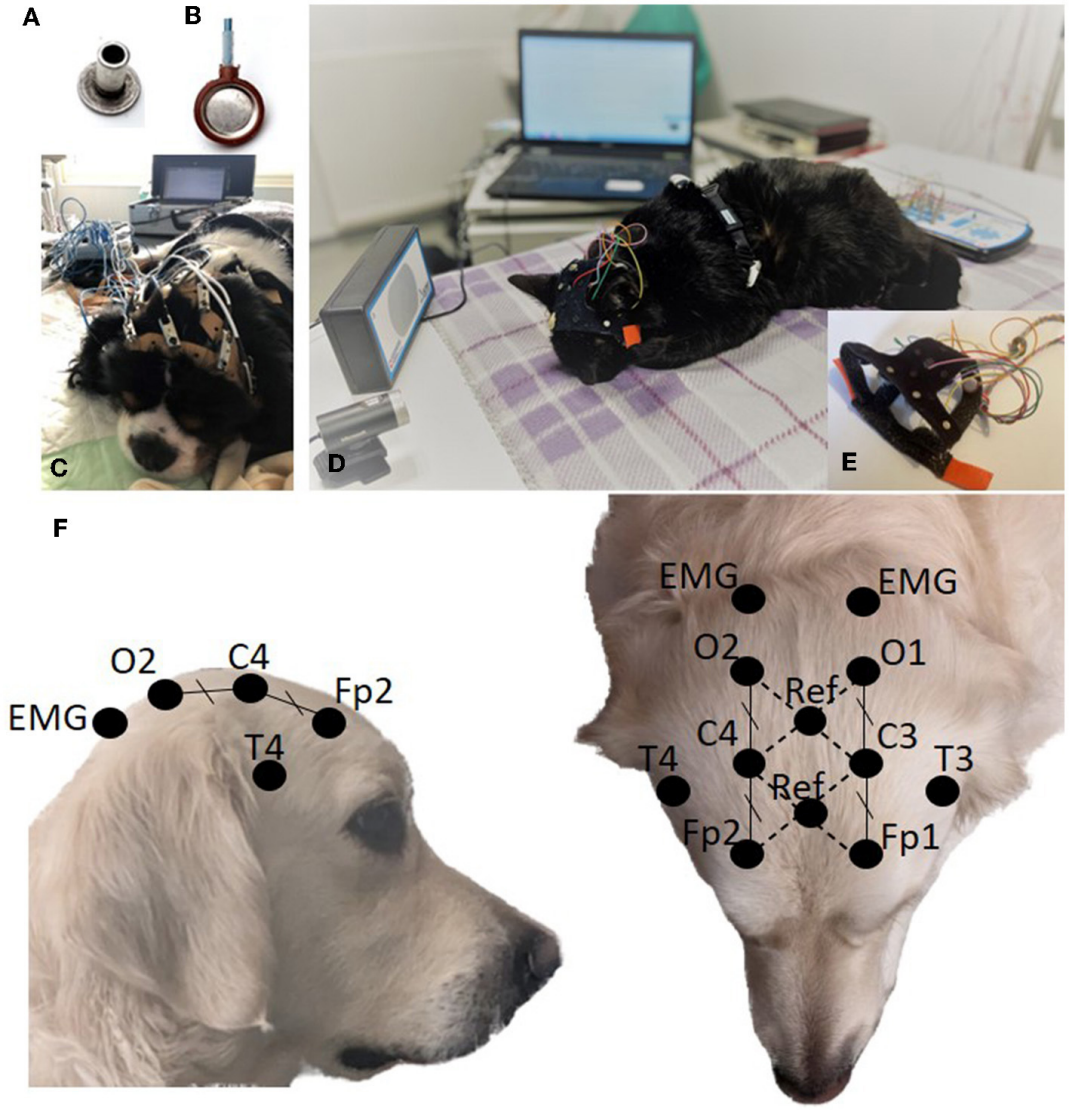
Stud electrode **(A)**. Cup electrode **(B)**. EEG acquisition setup using stud electrodes and elastic straps **(C)**. EEG acquisition setup using cup electrodes and PetCap^®^ **(D)** with details of electrodes coated with conductive paste and inserted into the system **(E)**. Electrode positioning **(F)**.

Human guidelines recommend the use of surface electrodes for routine EEG, either gold or Ag/AgCl cup gel electrodes or electrode caps (5, 6). As electrode caps are designed for human use, we opted for cup or stud surface electrodes, the latter being equivalent to non-wired cup electrodes. compatible with holding systems adapted to the animal's head. We compared unwired stud Ag/AgCl electrodes (Preborn, M.E.I, La Farlède, France; Figure 1A) and wired cup Ag electrodes (NE-112A, Nihon Kohden^®^, Tokyo, Japan; Figure 1B). The former was used over an initial period of 18 months, secured to the animals' heads using elastic straps perforated every 1.5 cm, in which the electrodes were inserted and held in place by alligator clips of the electric cables (Figure 1C). The latter was used over a second phase of 14 months, when an electrode EEG cap designed for cats and dogs was available in seven sizes (PetCap^®^, Elyope, Saint-Maur-des-Fossés, France) (32) (Figures 1D, E).

### 2.4 Electrode position and skin contact

The placement of the eight electrodes, both on the elastic straps and the PetCap ^®^, was based on the proposals of Pellegrino and Sica (22) and James et al. (24). The frontal electrodes (Fp1/Fp2) were positioned caudal to the median canthi, on the external edge of the temporal line. The occipital electrodes (O1/O2) were placed on a transverse line between the mastoid processes at an equal distance from the midline as the frontal electrodes. The central electrodes (C3/C4) were placed halfway between the frontal and occipital electrodes in the transversal plan and at an equal distance from the midline as the frontal and occipital electrodes. The temporal electrodes (T3/T4) were placed at the base of the ear, just above the temporal crest (Figure 1F). Our acquisition software allows the use of 2 reference electrodes and calculates the electrical potential difference between them to eliminate the noise. These 2 reference electrodes were placed on the median line at the intersection of diagonals Fp1-C4 and Fp2-C3 for the first reference and at the intersection of diagonals C3-O1 and C4-O2 for the second (Figure 1F). The electrodes were placed on the unshaven head, as close as possible to the skin, parting the hairs. For stud electrodes, elastic straps were placed on the animal's head, then the electrodes were inserted symmetrically into the perforations of the straps. Conductive paste and gel (Ten20^®^, Weaver and Company, Aurora, CO, USA, and SignaGel^®^ Parker Laboratories, INC. Fairfield, NJ, USA) were then applied between the skin and the electrode. For cup electrodes, the electrodes were pre-inserted in the PetCap ^®^ and coated with Ten20^®^ before placing the headset on the animal and adding the SignaGel^®^, which reduced intervention time (Figure 1E).

Simultaneously, an electrocardiography (ECG) and respiratory recording were performed, with a thoracic electrode and a movement sensor held by a chest strap positioned behind the animal's front legs. Two electromyography (EMG) surface electrodes were placed on the anterior and dorsal regions of the neck muscles, posterior to the occipital electrodes, secured by the PetCap^®^ (Figures 1E, F).

### 2.5 Recording

Electroencephalography recordings started after checking the impedance values of the electrodes and monitored in real-time, allowing for electrodes adjustments if necessary.

The camera was positioned above and facing the animal and it was repositioned if the animal moved (Figure 1D).

Intermittent Photic Stimulation (IPS) was performed at the beginning of the examination when the animal was lying down in a state of wakefulness. The lamp was positioned at eye level, 30 cm away from the animal and the program followed a program that increased the frequency of light flashes: 3–5–7–10–13–15–17–20–25–30–35–40–45–50 Hz, with 10 s duration and 5 s pauses between frequency changes (Figure 1D). If the patient fell asleep during the photic stimulation, this test was repeated at the end of the EEG examination after the patient woke up. Other stimulations such as noise and meal were carried out based on potential seizure triggers reported by the owner and the clinician.

The animals were not stimulated to promote rest for at least 20 min. If the patient fell asleep during the recording, the waking phase after the nap was recorded for a minimum of 5 min.

The operator annotated the recorded trace with as much information as possible concerning the events that may occur during the examination, in the environment or specific to the patient.

### 2.6 Interpretation

All recordings were visually reviewed by three authors SBe, CE, and EL during joint reading sessions in order to obtain a consensus. The settings used for interpretation were the same as for acquisition, but could be modulated to aid pattern discrimination. Artifacts, physiological rhythms, and paroxysmal events were listed. Wakefulness was identified on the EEG by visualization of a low voltage fast activity background disturbed by eye and body movement artifacts and muscle contractions (EEG and EMG channels). Drowsiness was identified by visualization of a low voltage fast EEG activity background, with alpha rhythms (8–12 Hz) or theta rhythms (4–7 Hz), fewer eye and body movement artifacts, less muscle tone and more regular breath (respiratory movement sensor). Non-rapid eye movements (Non-REM) sleep was identified by the occurrence of medium and high voltage delta (1–4 Hz) activity and/or sleep spindles (waves with a frequency of 12–16 Hz) in the EEG, no eye and body movement artifacts, regular respiration and decreased muscle tone. REM sleep was identified by visualizing of a low voltage fast activity on EEG, weak amplitude EMG but disturbed during facial or leg twitches and myoclonic jerks, irregular respiration (respiratory belt) and heart beat (ECG) (33–36). Paroxysmal events noted included spikes, polyspike-complex, spikes-and-slow-waves-complex, polyspikes-and-slow-waves-complex, sharp waves, triphasic waves, and slow waves (37, 38).

### 2.7 Statistics

In the absence of normal distribution, non-parametric tests were used. Descriptive statistics are presented as median [1st quartile−3rd quartile]. Quantitative variables between groups were compared by the Mann–Whitney–Wilcoxon test (for two groups) or the Kruskall–Wallis test and Dunn-Bonferroni *post-hoc* test (for more than two groups). The distributions of multiple groups were compared using the chi-square test of homogeneity, with some groups aggregated if numbers were insufficient and with Yates' continuity correction if necessary. Results were considered statistically significant at *P* < 0.05. All statistical tests were carried out using R software (4.2.1). The plots were generated using the R package ggplot 2 (39) and Microsoft Excel^®^ (Microsoft Corporation One Microsoft Way, Redmond, WA, USA). We used a ratio expressed in percentage, called “readable percentage” calculated with the time corresponding to the number of 20-second pages readable divided by the total recording duration in minutes. Indeed, if more than half of the 20-second page was uninterpretable due to artifact, i.e., whose amplitude causes overlapping of the recording channels, whatever the montage used (referential or bipolar) at the 7 μV/mm setting, or whose frequency overloads the visualization of physiological rhythms, the page was considered unreadable. In addition, EEG recording was stopped during impedance checks and restarted afterwards. Pages during these impedance checks were also counted as unreadable.

## 3 Results

### 3.1 Population

Two hundred and thirty EEGs were investigated between October 2019 and July 2022.

The first 80 recordings were used to develop the protocol, specifically to establish the positioning of the elastic bands for achieving a symmetrical setup during the recordings, validate the electrode positions with PetCap^®^, choose the types of surface electrodes, select the contact gels and equip the EEG device with video and synchronized photic stimulation lamp.

The following 150 recordings were included in the study involving 140 animals, 101 made on dogs and 49 on cats.

Ninety-four dogs were recorded with 88 dogs recorded once, five dogs twice and one dog, three times. Forty-one dog breeds were represented, 18 dogs were mixed breed. The head conformations of dogs were classified into three categories: dolichocephalic, with a very elongated skull, brachycephalic, with a very flat face, and mesocephalic, close to the primitive type (40) (Table 1). They were 43 female and 51 male dogs, ranging in age from 4 months to 17 years [4.3 years [1.8–7.6]], with weight ranging from 2.6 to 64.6 kg [17.2 kg [8.9–25.45]]. Five dogs were healthy dogs brought in by their owners who were veterinary students involved in the study, while all other dogs were presented by their owners for neurology consultations. Eighty dogs had a history of at least one paroxysmal episode in the 6 previous months, either typical epileptic seizures or less characteristic episodes such as tail chasing, myoclonus, episodic stiffness or ataxia, compulsive licking, fly biting, episodic collapses, trance-like episodes, jaw chattering episodes, episodic drooling, episodic chewing, episodic aggression, episodic polypnea or episodic movement disorders. Nine dogs had a confusional state or signs of vestibular impairment but no paroxysmal event.

**Table 1:** Distribution of dog breeds.

**Mesocephalic (68 dogs)**
American Staffordshire Terrier, Australian Shepherd (2), Australian Shepherd cross-breed, Basset Hound, Beagle, Beagle cross-breed, Bearded Collie, Beauceron cross-breed, Bernese Mountain dog, Bouvier des Flandres, Brittany Spaniel cross-breed, Bull Terrier cross-breed, Cavalier King Charles (3), Chihuahua (2), Cocker Spaniel, Coton de Tulear, Dutch Shepherd, Eurasier, Great Dan, German Shepherd, German Spitz, Golden Retriever (4), Irish Setter (2), Jack Russell (6), Labrador (4), Malinois (3), Malamute cross-breed, Maltese, Newfoundland cross-breed, Parson Russell Terrier, Rottweiler, Shiba Inu, Siberian Husky (3), Welsh Corgi Pembroke cross-breed, West Highland White Terrier, White Swiss Sheperd, Yorkshire Terrier (6), Yorkshire Terrier cross-breed, indeterminate cross-breed (5).
**Brachycephalic (11 dogs)**
Continental Bulldog, French Bulldog (6), Boxer, Carlin (2), Lhassa Apso cross-breed.
**Dolichocephalic (15 dogs)**
Border Collie (6), Border Collie cross-breed (4), Dachshund, Wirehaired Dachshund, Doberman, Italian Greyhound, Podenco.

Forty-six cats were recorded, with 43 cats recorded once and 3 cats twice. Six cat breeds, 1 mix breed and domestic shorthair cats were recorded [Bengal, Birman (2 cats), Devon Rex, Norwegian, Persian, Ragdoll (2 cats), Mix breed Main Coon, Domestic Shorthair (37 cats)]. They were 23 female and 23 male cats, ranging in age from 7 months to 17 years [3.5 years [1.4–7.9]], with weight ranging from 700 to 7.4 kg [4 kg [3.15–4.8]]. Forty cats had a history of at least one paroxysmal event, either typical epileptic seizures or less characteristic episodes such as rolling skin, tail chasing, scratching, episodic aggression, episodic growling, episodic vocalization, compulsive licking, trance-like episodes, episodic stiffness or ataxia, possible REM sleep disorder or episodic movements disorders. Six cats had a confusional state or signs of vestibular impairment but no paroxysmal event.

### 3.2 Validation of unsedated EEG feasibility

Ninety-four percent (141/150) of the EEG recordings were carried out without the use of sedative medication. Two dogs underwent EEG after Magnetic Resonance Imaging (MRI) or Brainstem Auditory Evoked Response (BAER) and were under sedation, while three cats and one dog received preventive premedication due to their aggressiveness (dexmedetomidine/butorphanol for one cat and gabapentin for the others). Additionally, three restless dogs were sedated to minimize movement and breathing artifacts (dexmedetomidine). The results presented here pertain to EEG without tranquilization, i.e., 95 EEGs performed on dogs and 46 EEGs performed on cats.

Sixty-five dog EEGs and 28 cat EEGs were performed in the presence of the owner. Treats were used to keep the dogs occupied if they became agitated during the helmet fitting. All dogs kept the EEG system on their head and one cat initially removed the system which was reinstalled. Despite gentle movements, lying down in various position (sternal or lateral recumbency), standing up, or shaking, the electrodes remained attached with good electrode-skin contact and impedances. In some cases, a strip of elastic band (Vetrap™) was applied preventively to secure the mounting system if the animal seemed agitated before recording. No dermatological reactions were reported following the use of Ten20^®^ and SignaGel^®^. Gel was removed using dry shampoo and, if necessary, supplemented with wet shampoo at home by the owner, like in human use.

Sixty-four dog EEGs were performed using stud electrodes, and 31 dog EEGs were conducted using cup electrodes. Twenty-four cat EEGs were conducted with stud electrodes, and 22 cat EEGs were performed with cup electrodes.

Photic stimulation was achieved for 42 dog EEGs and 27 cat EEGs, as the recording device was not initially equipped for it at the start of the study. Light stimulation was not performed in cases of non-convulsive status epilepticus diagnosed by EEG (4 EEGs).

Other stimulations were carried out according to history provided by owners to trigger seizures, including food (five dogs) and sounds (three dogs and one cat) such as clapping of hands, sound of keys, sound of crumpled paper and sound of crushed plastic bottles.

**Figure 2:**
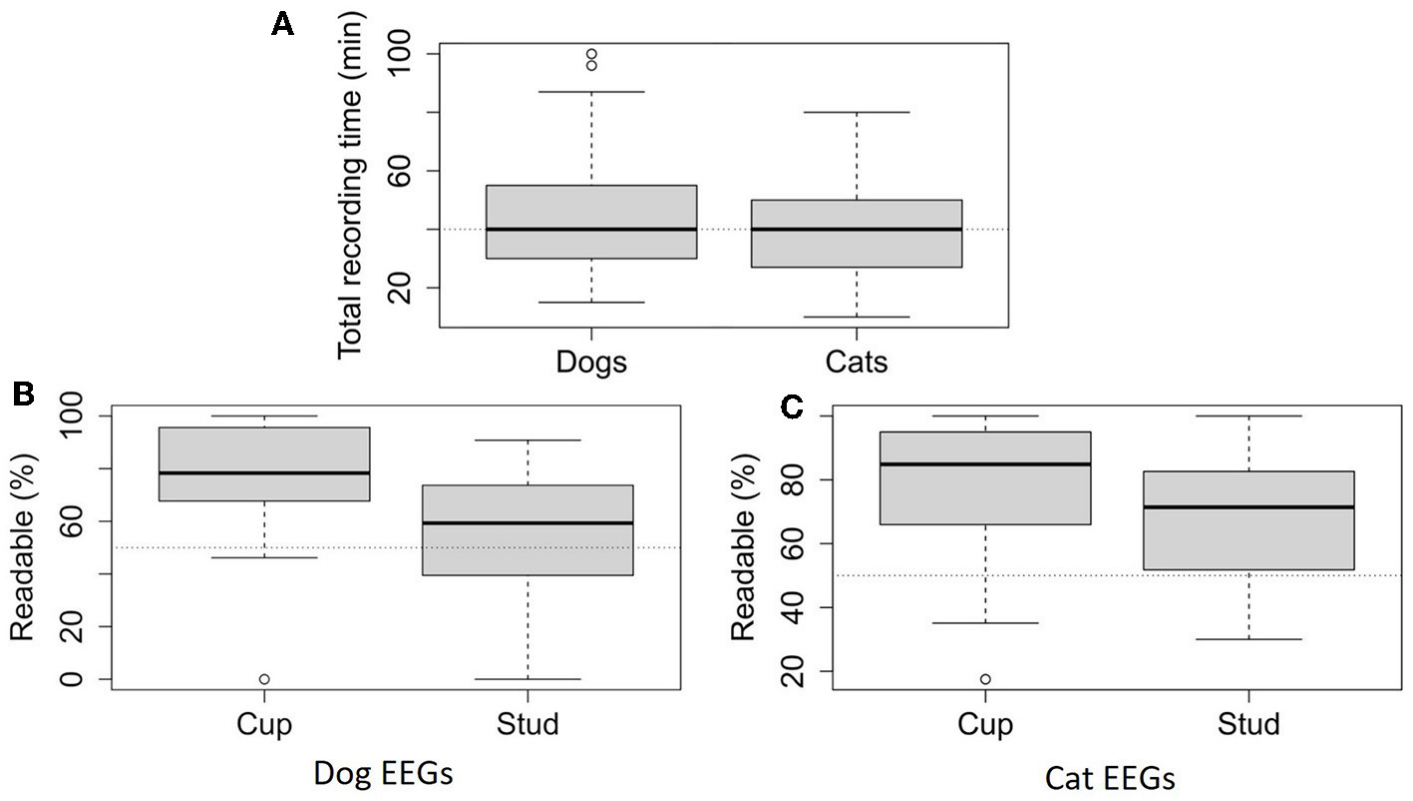
Total recording time for dog EEGs (95) and cat EEGs (46) **(A)**. Readable percentage calculated with the 20-second pages that are undisturbed by artifacts for more than half of their duration over the total recording time depending on electrode type, for dog EEGs (64 EEGs with stud electrodes and 31 EEGs with cup electrodes) **(B)** and cat EEGs (24 EEGs with stud electrodes and 22 EEGs with cup electrodes) **(C)**.

Median EEG recording time was 40 min for the two species [Dogs: 40 min [30–55]; Cats: 40 min [27–49]] (Figure 2A). Six patients had recording time of < 20 min, with 4 due to time constraints related to the functioning of the service, 1 where the diagnosis was immediately established based on EEG findings and 1 due to excessive agitation. Twenty-six animals had a recording lasting more than 60 min, 12 were sleeping deeply, nine were restless and we had to wait for the animal to calm down to have a readable EEG trace, and five others to maximize the chances of recording seizures.

### 3.3 Validation of the technical quality of the EEG recordings

#### 3.3.1 Impedance

**Figure 3:**
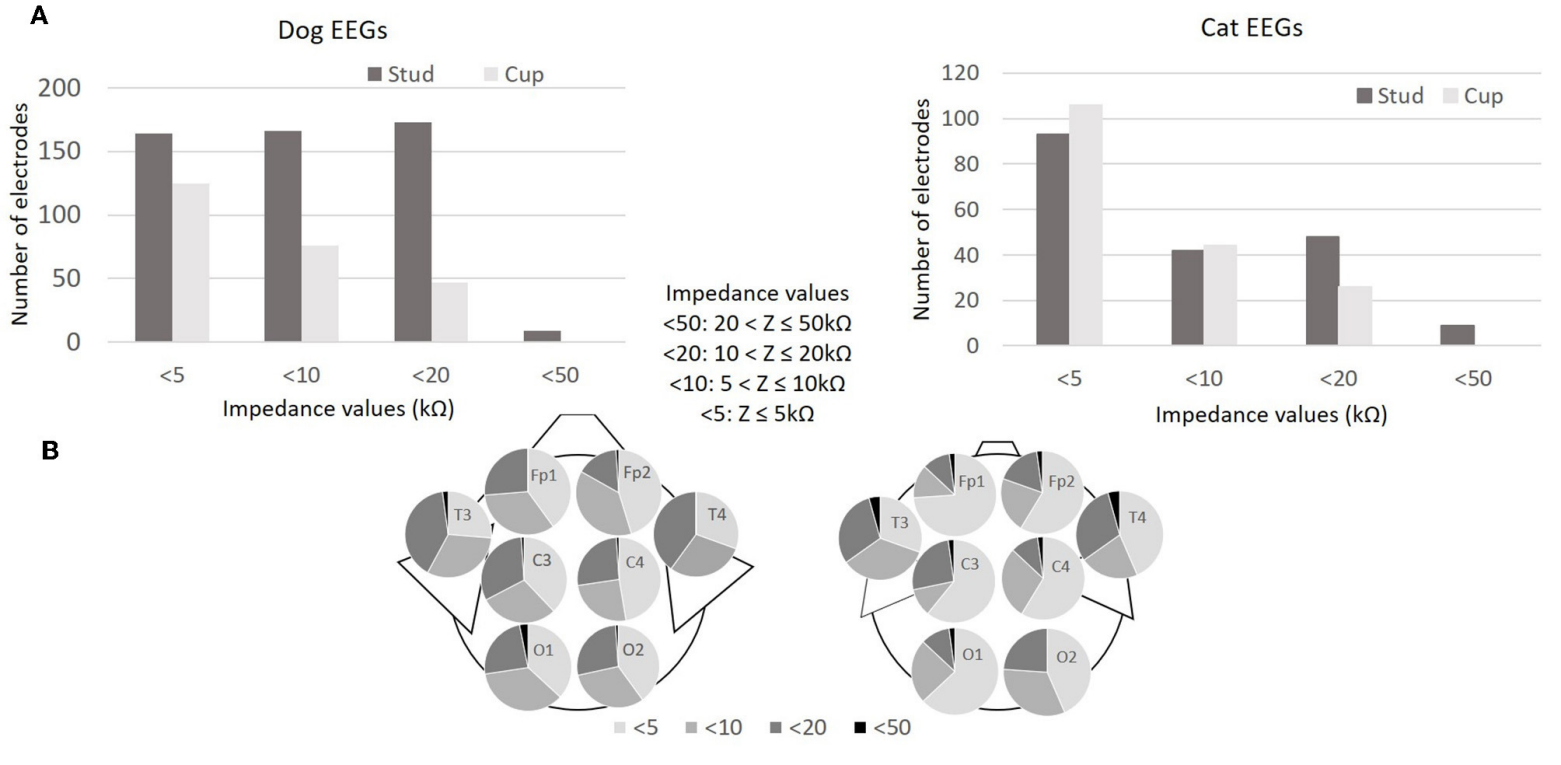
Impedance values (kΩ) of the 760 electrodes during the 95 dog EEGs (95x8 electrodes) and impedance values of the 368 electrodes during the 46 cat EEGs (46x8 electrodes) **(A)**. Distribution of impedance values (kΩ) according to electrode position in 95 dog EEGs and 46 cat EEGs **(B)**.

Electroencephalography software gives intervals of values of the impedance measurements (Z) for each electrode: Z > 100 kΩ (written >100 on the software), 50 < Z ≤ 100 kΩ (written < 100), 20 < Z ≤ 50 kΩ (written < 50), 10 < Z ≤ 20 kΩ (written < 20), 5 < Z ≤ 10 kΩ (written < 10), Z ≤ 5 kΩ (written < 5). All impedance values recorded were ≤ 50 kΩ (indicate < 5 or < 10 or < 20 or < 50 by the software). In dog EEGs, 98.8% (751/760) of the impedances were ≤ 20 kΩ (indicated < 5 or < 10 or < 20 by the software) and, in cat EEGs, 97.6% (359/368), regardless of the types of electrode used. Impedances ≤ 10 kΩ (indicated < 5 or < 10 by the software) were observed in 81 and 85.2% of dog and cat EEGs, with cup electrodes, and in 64.5 and 70.3% with stud electrodes (Figure 3A). Comparing the numbers in the < 5 and < 10 impedance groups with those in the < 20 and < 50 groups by electrode type (stud or cup), we concluded that cup electrode impedances are lower than stud electrode impedances (dog EEGs: x-squared = 121.32, df = 1, *p* < 0.001; cat EEGs: x-squared = 10.856, df = 1, *p* < 0.001; Figure 3A). Impedances are not homogeneous, depending on electrode positioning in dog and cat EEGs (dog EEGs: x-squared = 26.228, df = 14, *p* = 0.024; cat EEGs: x-squared = 33.97, df = 14, *p* = 0.002; Figure 3B).

#### 3.3.2 Montages and isosynchrony

**Figure 4:**
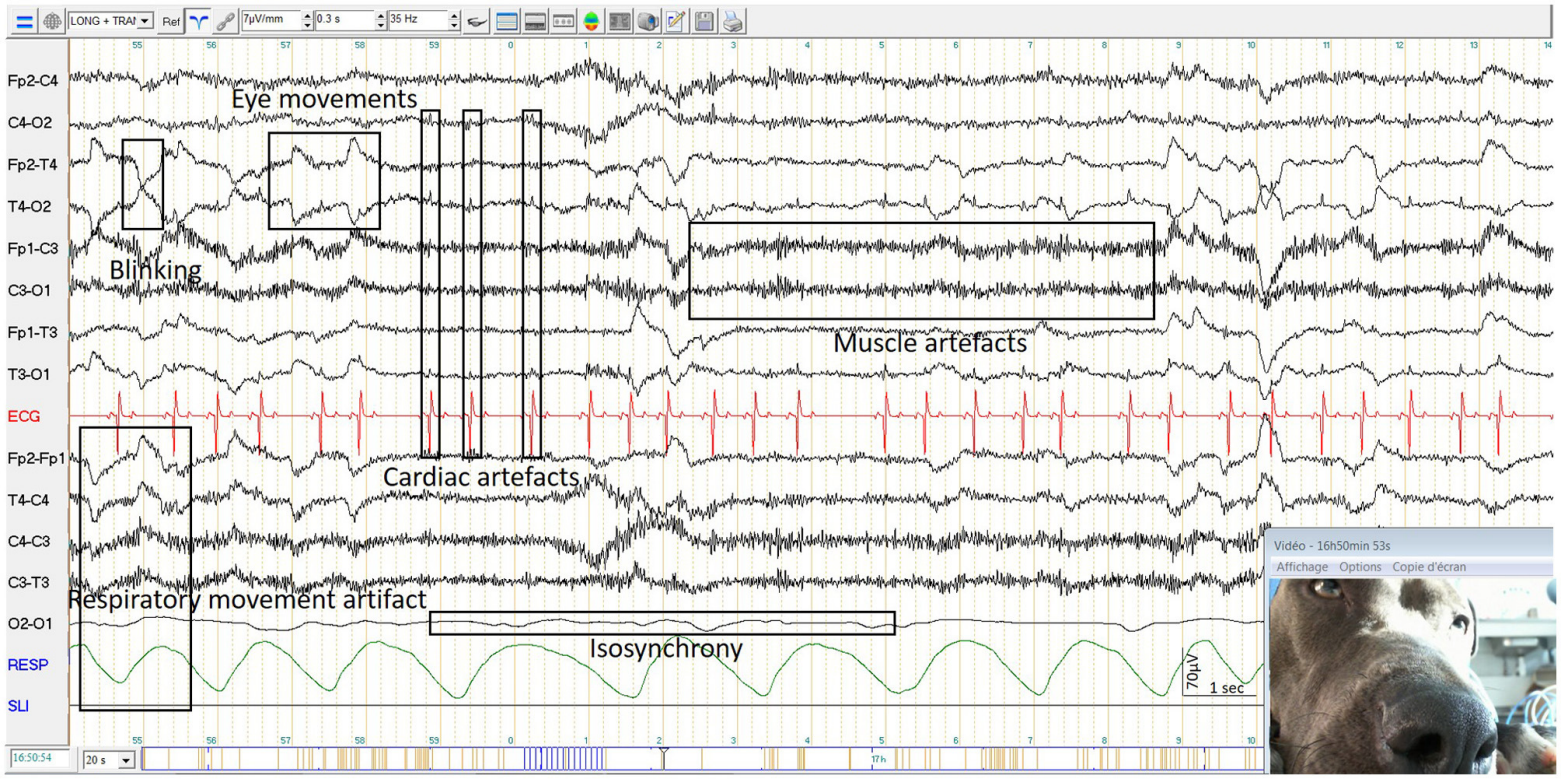
Examples of isosynchrony and physiological artifacts in a dog EEG.

We define “usable channels” as channels without isosynchrony, that appears on the EEG trace as a flat line (Figure 4). For dog EEGs, in the longitudinal montage, 93.4% (478/512) of the channels were usable with stud electrodes, and 94.7% (235/248) were usable with cup electrodes. In the transverse montage, 95.6% (306/320) of the channels were usable with stud electrodes, and 94.8% (147/155) with cup electrodes. For cat EEGs, in the longitudinal montage 83.3% (160/192) of the channels were usable with stud electrodes and 93.2% were usable (164/176) with cup electrodes. In the transverse montage 79.2% (95/120) of the channels were usable with stud electrodes, and 97.3% (107/110) were usable with cup electrodes. By adding the values of the usable channels of dogs and cats in longitudinal montage with cup electrodes (235 + 164 = 399) and dividing by the total number of channel of dogs and cats in longitudinal mounting with cup electrodes (248 + 176 = 424) we obtained the proportion of usable channels which was in percentage 94.1%. The percentage of isosynchrony in the longitudinal montage with cup electrodes for both dog and cat combined was therefore 5.9% (100–94.1%).

**Figure 5:**
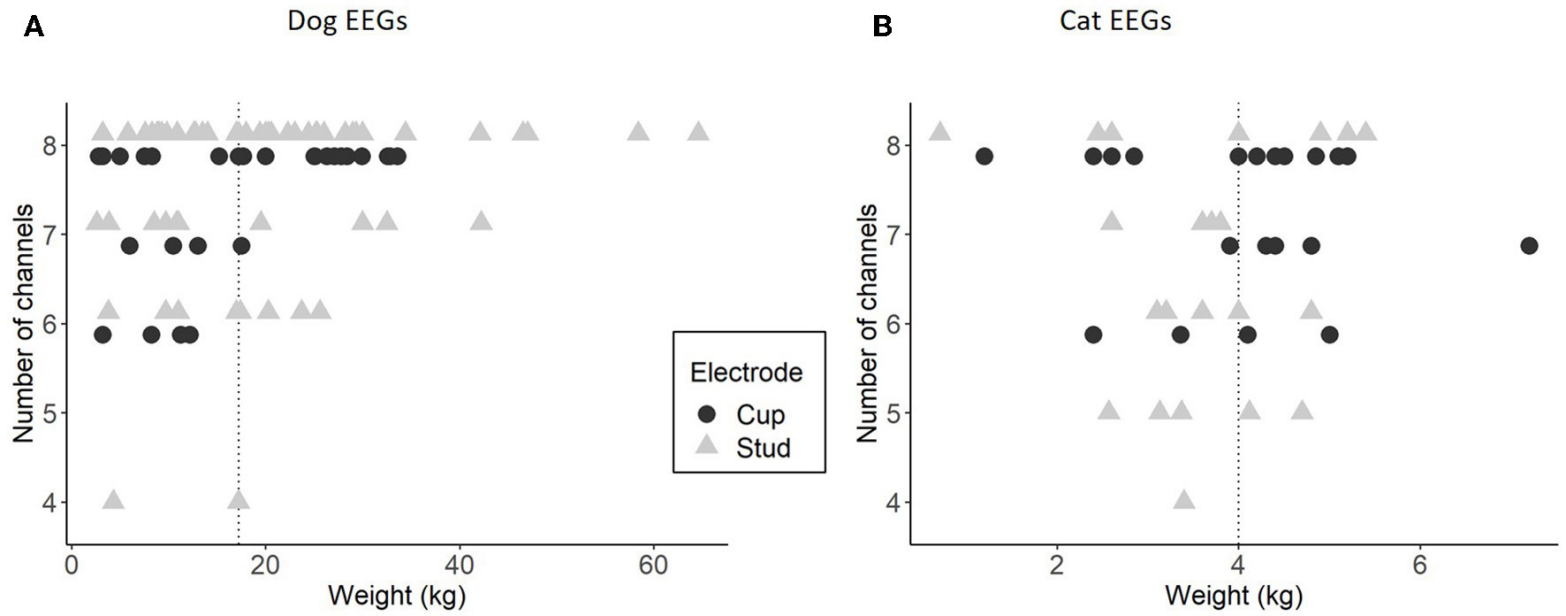
Relationship between the weight of the animals and the number of usable channels in longitudinal montage, for dog EEGs with stud (64 EEGs) and cup (31 EEGs) **(A)** and for cat EEGs with stud (24 EEGs) and cup (22 EEGs) **(B)**. The dotted line represents the median of the weights in each species.

The longitudinal montage with eight usable channels was more easily set up in larger dog compared to small dogs (Figure 5A). This difference is less noticeable in cats, as the variation in weight and size is less significant (Figure 5B). The 3 EEGs with only four usable longitudinal channels were performed with stud electrodes on restless animals. One of these EEGs was repeated due to the animal's agitation, and the number of channels was eight during the second recording.

#### 3.3.3 Artifacts

We observed technical artifacts such as electrical environment artifacts, particularly when the amplifier box was positioned on a metal surface, as well as pop artifacts from the electrodes. Additionally, artifacts specific to the use of alligator clips with stud electrodes were observed when they were in contact with each other. We also observed artifacts related to people in the recording environment, including recording of the owner's cardiac pulse, petting the animal and contact with the wires, and movements in the room.

Some physiological artifacts observed were similar to those described in human medicine atlases, such as heartbeat, respiratory movements, muscle contractions and global movements (Figure 4). However, we observed also artifacts specific to dogs and cats, such as licking artifacts, ear movements and tail flapping movements.

Seventy-four point seven percent (71/95) of the dog recordings were readable for more than 50% of their duration up to 100%, 13.7% (13/95) of the recordings were readable for 25–50% of their duration and 11.6% (11/95) of the recordings were readable for < 25% of their duration. In the latter cases, the animal had behavioral problems, or the owners were intrusive, or stimulation techniques such as feeding generated a lot of artifacts. “Readable percentage” below 50% were mainly associated with the use of stud electrodes (20/24). Consequently, “readable percentage” were significantly higher in EEG recordings with cup electrodes than those with stud electrodes [stud electrodes: 59% [40–73]; cup electrodes: 78% [68–96]; *p* < 0.001; Figure 2B].

Nine dogs had 2 registrations, 4 due to their agitation and the others for medical follow-up. The median of readability percentages of the first recordings of agitated dogs was 16% [12–25], while that of the second recordings was 70% [61–75], suggesting the beneficial effect of habituation in some specific cases.

Eighty-two point six percent (38/46) of the cat recordings were readable for more than 50% of their duration up to 100%, 15.2% (7/46) of the recordings were readable for 25–50% of their duration, 2.2% (1/46) of the recordings were readable for < 25% of their duration. The main difficulties encountered were related to inappropriate contact between the alligator clips, which is accentuated by the small size of the cats' heads and to the owners' interventions. In cats, we found no significant difference between the “readable percentage” on EEG recordings with stud electrodes and those with cup electrodes [stud electrodes: 71% [52–82]; cup electrodes: 85% [68–94]; *p* = 0.0503; Figure 2C].

### 3.4 Validation of EEG interpretability

#### 3.4.1 Physiological rhythms

**Figure 6:**
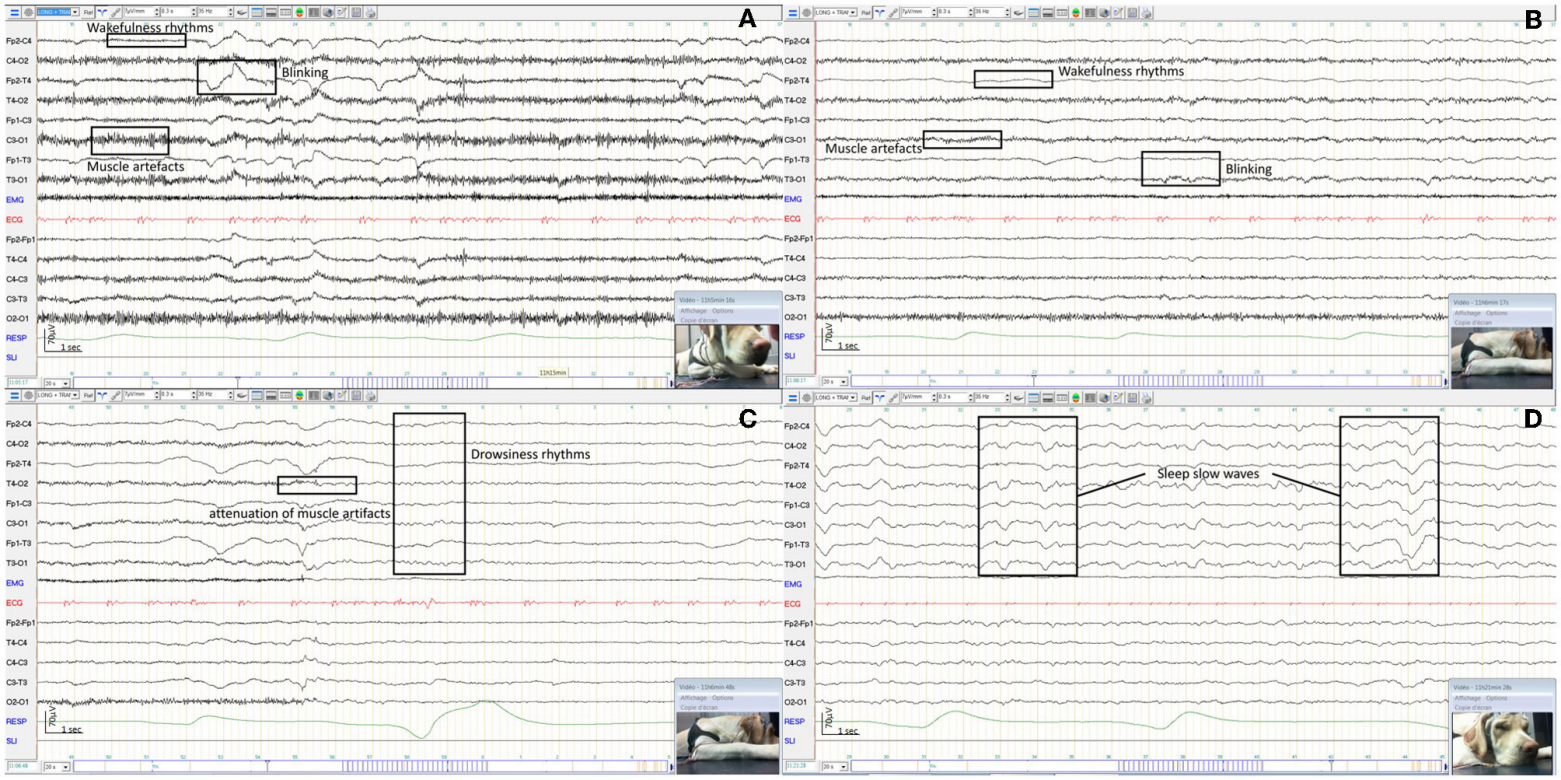
EEG of an awake dog showing a rapid low amplitude rhythm and artifacts from blinking and muscle activity with examples in boxes **(A)**. EEG of the same dog as before, awake and calm, showing a rapid low amplitude rhythm with reduced artifacts from blinking and muscle activity with examples in boxes **(B)**. EEG of the same dog as before, dozy, showing a 5 Hz medium amplitude rhythm **(C)**. EEG of the same dog as before, sleepy, showing 1−3 Hz high amplitude rhythms with examples in boxes **(D)**.

**Figure 7:**
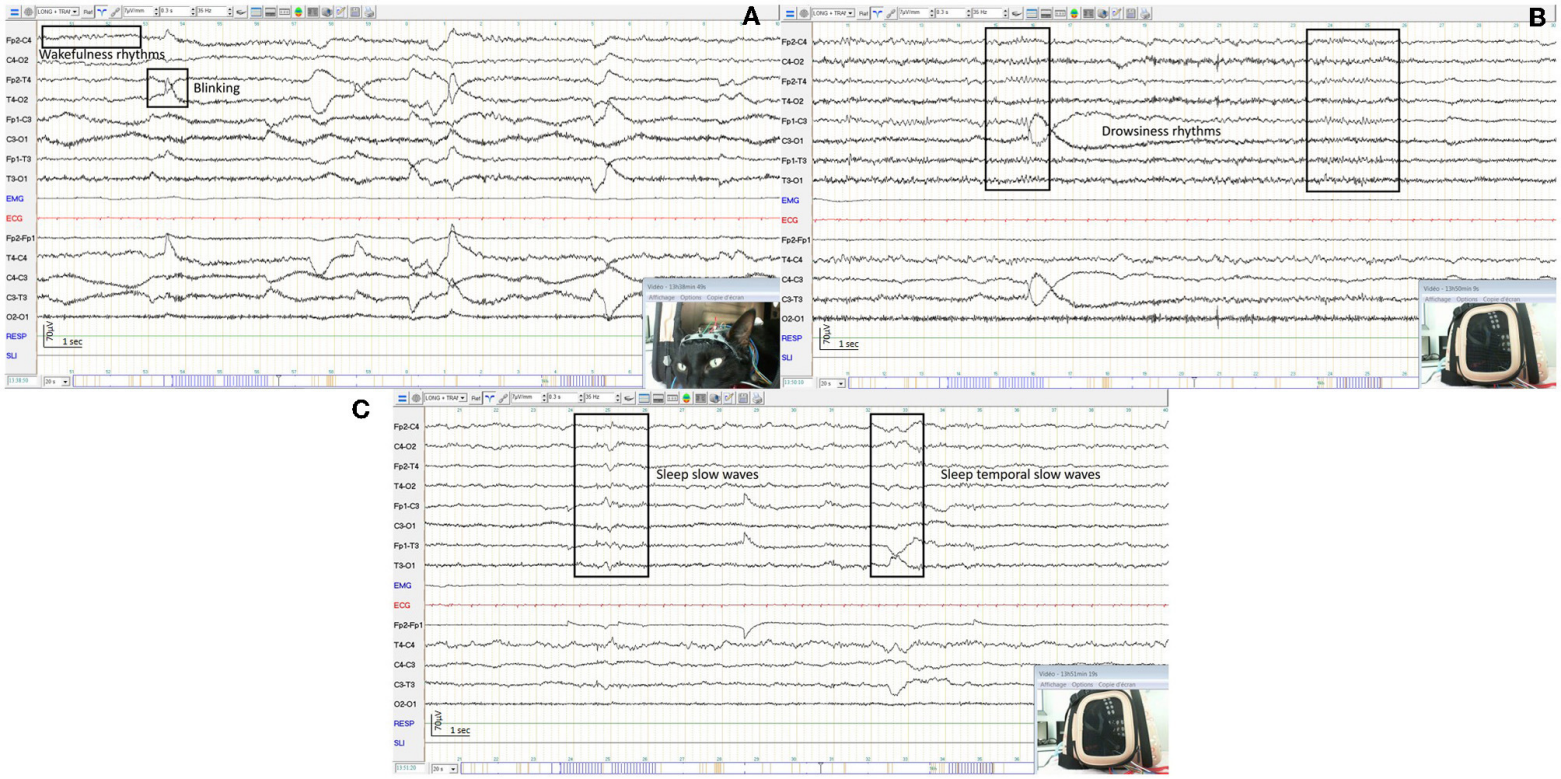
EEG of an awake cat showing a rapid low amplitude rhythm and artifacts from blinking and muscle activity with examples in boxes **(A)**. EEG of the same cat as before, dozy, showing a 7 Hz medium amplitude rhythm **(B)**. EEG of the same cat as before, sleepy, showing mixed theta and delta rhythms with examples in boxes **(C)**.

Physiological rhythms were observed in 98.9% (94/95) of dog EEGs including wakefulness rhythms (92/94) (Figures 6A, B), drowsiness (72/94) (Figure 6C), and sleep (46/94) (Figure 6D). For cat EEGs, physiological rhythms were observed in 91.3% (42/46), including wakefulness rhythms (41/42) (Figure 7A), drowsiness (33/42) (Figure 7B), and sleep (9/42) (Figure 7C). No physiological rhythms were observed in five recordings, probably due to the pathology of four animals (diffuse encephalopathy for one dog and one cat, a non-convulsive status epilepticus for two cats), and to technical reason in one cat (intact male with high impedance values possibly due to skin specificity).

**Figure 8:**
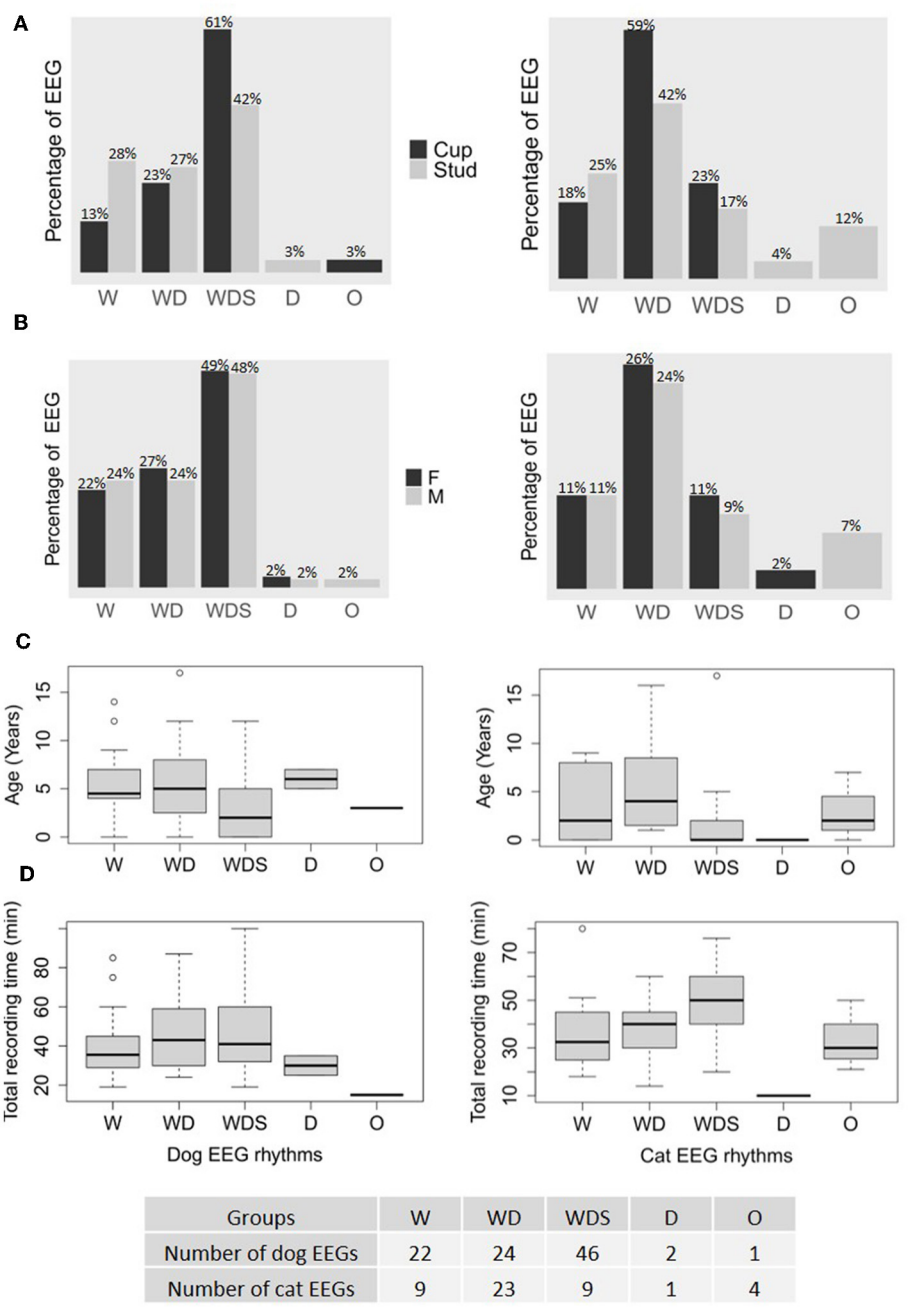
Rhythm distinctions (W, wakefulness only; WD, wakefulness and drowsiness; WDS, wakefulness, drowsiness and sleeping; D, drowsiness only; O, other than physiological states) in the recordings of 95 dogs (on the left) and 46 cats (on the right) detailed according to the type of electrodes used and expressed as a percentage of the total number of recordings made for each kind of electrode, cup and stud **(A)**, their sex **(B)**, their age **(C)**, and the total recording time **(D)**.

Five groups were determined based on the physiological rhythms observed: EEG with wakefulness rhythms only (W), EEG with wakefulness and drowsiness rhythms (WD), EEG with wakefulness, drowsiness and sleeping rhythms (WDS), EEG with drowsiness only (D), and other than physiological rhythms (O). The W group consisted of 22 EEGs from dogs and 9 EEGs from cats, the WD group had 24 dog EEGs and 23 cat EEGs, the WDS group had 46 dog EEGs and 9 cat EEGs, the D group had 2 dog EEGs and 1 cat EEG, and the O group had 1 dog EEG and 4 cat EEGs. The three groups W, WD and WDS were observed in both species of the study regardless of the two types of electrodes (Figure 8A). Dogs were more likely to sleep and cats to doze during recordings and this tendency was more pronounced with cup electrodes (Figure 8A). Physiological rhythms were observed in both males and females (Figure 8B) and they were easier to observe in younger animals compared to older ones (Figure 8C). The ages of dogs in the three groups W, WD and WDS were significantly different (dog EEGs: Kruskall-Wallis chi-squared = 8.32, df = 2, *p* = 0.02; cat EEGs: Kruskall-Wallis chi-squared = 6.48, df = 2, *p* = 0.04) with younger animals in the WDS group than in the W group (*p* = 0.03) and in the WD group (*p* = 0.03) in dog EEGs and with younger animals in the WDS group than in the WD group (*p* = 0.02) in cat EEGs (Figure 8C). Median recording time in W groups was 35.5 min [29–45] for dog EEGs and 32.5 min [25–44] for cat EEGs, in WD groups 43 min [30–58] for dog EEGs and 40 min [30–45] for cat EEGs, and in WDS groups was 41 min [33–59] for dog EEGs and 50 min [42–59] for cat EEGs (Figure 8D). The other two groups, D and O, involved only a few animals and were related to their respective disease.

#### 3.4.2 Stimulation tasks

##### 3.4.2.1 Intermittent photic stimulation

**Figure 9:**
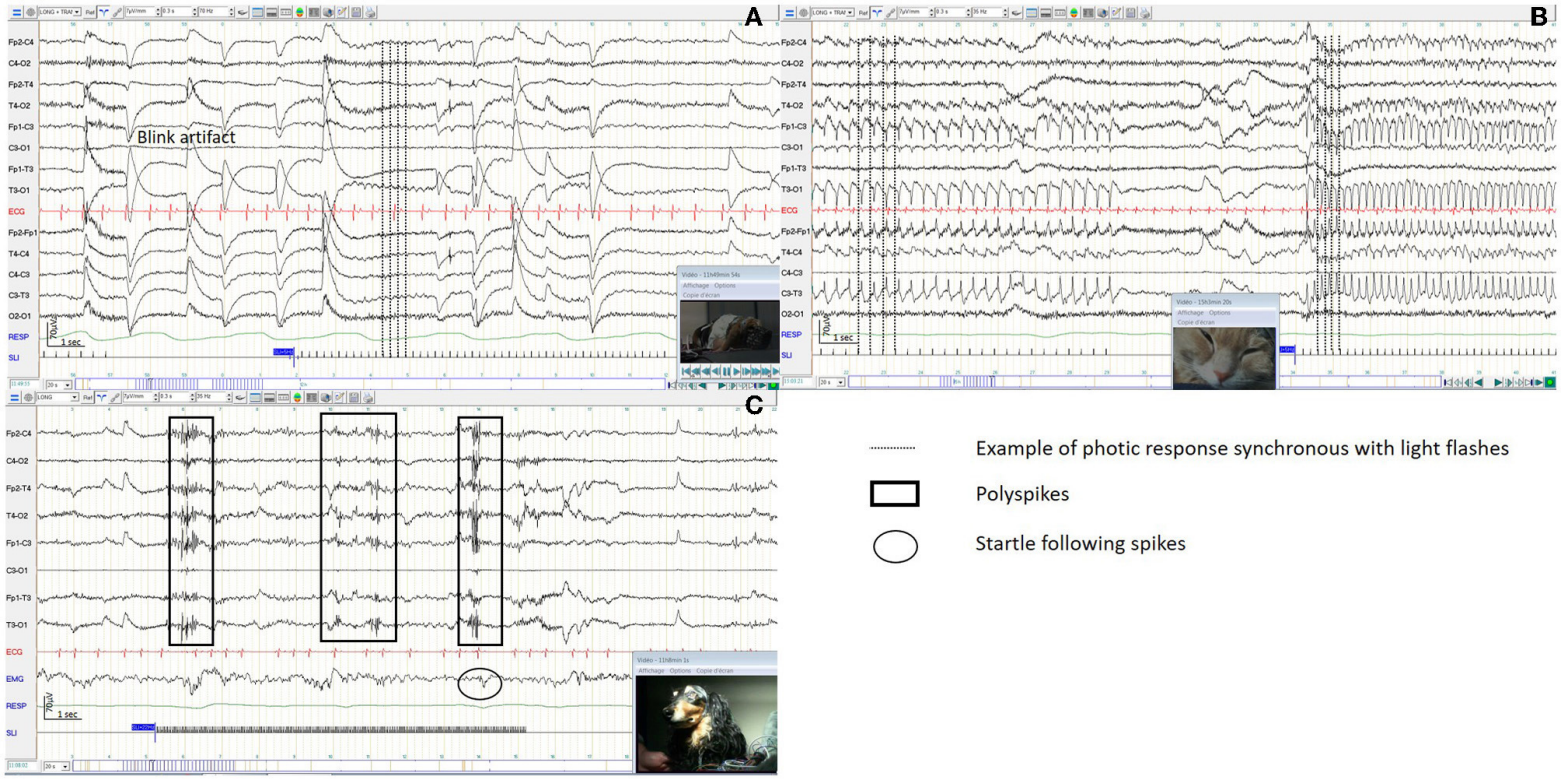
Reactivity to intermittent photic stimulation in a dog EEG **(A)**, in a cat EEG **(B)**, with polyspike complexes in a dog EEG **(C)**.

Photic response synchronous with light flashes were observed in 11% (5/45) of dog EEGs, with frequencies ranging from 3 to 10 Hz and in 85% (23/27) of cat EEGs, with frequencies ranging from 3 to 30 Hz, without any clinical signs (Figures 9A, B). Epileptic discharges were observed during IPS in 13% (6/45) of the dog EEGs (Figure 9C). For 5 dogs, clinical myoclonus was observed simultaneously with epileptic discharges, while for the remaining dog no clinical manifestation was observed. Four of these six dogs showed mild myoclonus during sleep and epileptic discharges on the EEG trace.

##### 3.4.2.2 Other stimulation tasks

During stimulation with food with five dogs, two dogs presented clinical paroxysmal event including one epileptic and one non-epileptic. During stimulation with sounds with three dogs and one cat, all presented clinical paroxysmal event. On the EEG, epileptic discharges were observed simultaneously with clinical paroxysmal event in one dog and in one cat.

#### 3.4.3 Electro-clinical interpretation

**Figure 10:**
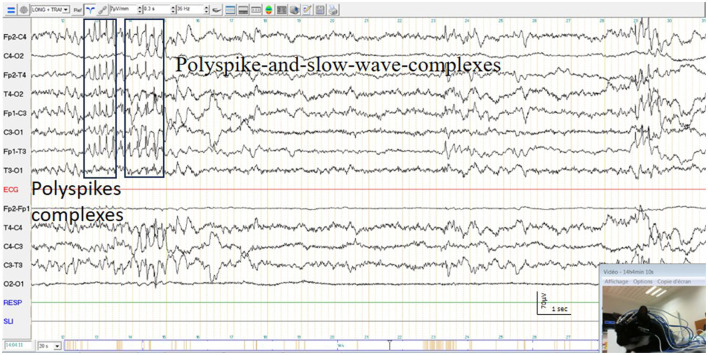
Polyspike complexes and polyspike-and-slow-wave-complexes and simultaneous twitching of a cat's face.

No animal exhibited generalized tonic-clonic epileptic seizure during EEG recordings. However, 37 animals showed symptoms suggesting epileptic seizure, like myoclonus localized on the face, limb or trunk, with a wide range of intensity such as tremors, sudden movements or startles or alterations in consciousness or neurobehavioral signs as shivering and polypnea (41). Among these animals, 75.7% (28/37) exhibited concomitant pathological graphoelements, with 59.5% (22/37) displaying epileptic patterns (37) (Figure 10) and 16.2% (6/37) showing other pathological graphoelements, such as triphasic waves and periodic discharges, suggestive of encephalopathy. Sixteen point two percent (6/37) of animals showed paroxysmal manifestations not followed by EEG abnormalities, ruling out an epileptic cause. Eight point one percent (3/37) of animals exhibited clinical symptoms, but artifacts on the EEG trace hindered interpretation. Additionally, 9 EEGs showed epileptic or seizure patterns without any clinical signs during electrical events.

Among the 136 non control and non-tranquilized animals, 27.2% (37/136) presented clinical signs during the examination and 25% (34/136) of the EEGs provided diagnostic information. Furthermore, 6.6% (9/136) of the EEGs showed epileptic or seizure patterns suggestive of epilepsy without clinical signs (37). Thus, EEG was informative in 31.6% (43/136) of the patients, despite the inclusion of a highly variable population (Table 2).

**Table 2:** Correlations between clinical and electrical patterns observed during the EEG according to literature (37).

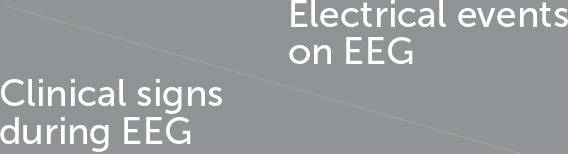	**Epileptic or seizure patterns**	**Other electrical events (Periodic discharges and triphasic waves)**	**No electrical event**	**Equivocal electrical events (due to artifacts)**
Myoclonus (head or body, or limb)	7 dogs 7 cats	1 dog	1 dog 3 cats	1 dog 2 cats
Alterations in consciousness	3 cats	2 dogs 3 cats		
Ptyalism +/- licking	4 dogs 1 cat			
Polypnea + shivering			2 dogs	
No clinical signs	5 dogs 4 cats			

## 4 Discussion and conclusion

The aim of this work was to propose a method that would facilitate the use of EEG in veterinary practice for routine use, adapted to specific constraints of the animal while respecting its wellbeing, so as to be able to observe brain function and disorders in the most physiological situation possible. We have succeeded in performing EEG examinations on 150 dogs and cats of various sizes and conformations in a clinical setting, without the need of anesthesia in 94% of cases. The cats were particularly calm during the examination, and the fact that they could remain in their open or covered transport box was beneficial to their relaxation. The presence of the owner and the possibility of lying down in their own basket helped the dogs a lot to relax. For the most agitated animals, the owner sometimes lay down next to his animal or took it on his lap. Managing ambient noise was particularly important, with some preferring silence and others a monotone background noise like that of a discussion. We also took care to be few in number in the room, placing ourselves far from the animal, in a seated, calm position and looking away from the animal and oriented toward the camera screen, being the least interventional as possible and patient.

Our study allowed to describe the performance of EEG without need for anesthesia or sedation, medical training or voluntary recruitment (24, 35, 42–44), in a large sample of dogs and cats. To our knowledge, no study on EEG without anesthesia in healthy or diseased cats has been conducted prior to this one. The use of surface electrodes and the ability to mount them on a holding system gave us a real advantage over the use of needles in being able to offer this examination to any animal showing signs of encephalic dysfunction, whatever the temperament of the animal and with the full confidence and endorsement of the owner. The latter could even assist with the EEG, reassuring their animal, helping with the setup of the system on animals that exhibited a particularly wary attitude toward the veterinarian, providing insights into the triggering elements of the seizures, and validating the concordance of the seizures observed during the examination and at home.

Surface electrodes are non-invasive, comfortable, provide high-quality EEG examinations and are widely used in human medicine. We therefore opted for surface electrodes rather than needle electrodes, but we had to consider a holding system to enable them to be positioned on the animal's head. This had to be able to ensure symmetrical and constant positioning in relation to the bony relief of the animal's skull, whatever its conformation. We tested 2 holding systems, each compatible with 1 type of surface electrode stud or cup. One consisted of elastic straps placed around the animal's head. These straps were perforated every 1.5 cm, enabling the stud electrode insertion holes to be selected according to the bone relief. The other consisted of a system to be threaded through the animal's head, and offered in 7 sizes to adapt to the varying conformations of the animals. The cup electrodes were fitted with covers so that they could be clipped onto the cap. This enabled us to compare two approaches: the first with a system that allows free placement of the electrodes, but whose installation is tedious, and the second with a more constrained system, but quick installation. The second system is similar to the pre-wired caps available for humans.

We were concerned to obtain good quality examinations to be able to interpret the tracings as accurately as possible. To achieve this, we used evaluation criteria and ensured that the quality of the tracings obtained was equivalent to that of other methods used in veterinary medicine. We also checked that this quality was in line with the recommendations published for the performance of EEGs in human medicine (2–6).

Impedances obtained were below 20 kΩ in 98.8% of dog EEGs and in 97.6% of cat EEGs, with better results with cup electrodes, which obtained impedances below 10 kΩ for over 80% of electrodes. These impedance values align with those reported in literature, ranging from 5 to 30 kΩ for needle electrodes in dogs (22–24, 44–46) and cats (47), and 5 to 15 kΩ for cup electrodes (23, 35) and with human medicine recommendations that impedance values for surface electrodes be below 10 kΩ (4, 6).

The derivations observed in longitudinal montage are determined for each hemisphere between the frontal and central, central and occipital, frontal and temporal and temporal and occipital electrodes, and are referred to as channels. The usable channel rate exceeds 93% for both species, the other channels being affected by isosynchrony linked to the proximity of the electrodes or to gel diffusion between two electrodes. This isosynchrony rate of 5.9% is close to the isosynchrony rate of 5.5% obtained with needle electrodes on dog EEG, with the same observation that isosynchrony was more frequent in smaller dogs (45). We have not found incidence values for isosynchrony in human medicine, but it is well-described. In medical research, dry electrodes were developed to eliminate the need for gel to avoid the problem of isosynchrony and to save time. Regardless of the technologies used, the impedances of these dry electrodes are higher than those of gel electrodes, and for some, especially contactless electrodes, the very weak electrical signal must be amplified directly at the electrode level, which makes them heavier and bulkier (48–50). In human medicine, gel electrodes remain the gold standard (6, 50). Dry electrodes are less studied in veterinary medicine. Polymer electrodes with a silver/silver chloride (Ag/AgCl) coating and gold-plated metal electrodes covered with poly(3,4-ethylenedioxythiophene) polystyrene sulfonate (PEDOT:PSS) have been studied as ECG sensors on dogs (51, 52). PressOn™ EEG electrodes and spring-loaded dry EEG electrodes have been used in a unpublished study, but the quality of the EEG signals obtained remains inferior to those recorded by needle electrodes in the same study (53). Hence, we suggest the utilization of gel electrodes in veterinary medicine while closely monitoring the potential advancements that the research and development of appropriate dry electrodes might yield.

We aimed to evaluate the impact of artifacts, which are inevitably more prevalent during EEG recordings without anesthesia. Consequently, we sought to clearly identify these artifacts, assess their extent of influence on the trace, and determine the required examination duration to obtain a 20-minute interpretable trace, in accordance with recommendations for routine EEG in human medicine (6). We have clearly recognized similar technical and physiological artifacts as described in human (38) and veterinary medicine (23, 45), in addition to specific ones originating from the examination situation without anesthesia and stimulation techniques such as food intake. The detailed description of these artifacts remains to be published in a future article. However, despite these artifacts, 75% of dog EEGs and 83% of cat EEGs were readable for more than 50% of their duration, with a median recording time of 40 min in both species. EEG tracings longer than 40 min can therefore provide sufficient data to be medically relevant with minimal impact from artifacts.

The recognition and identification of normal physiological rhythms of wakefulness, drowsiness and sleep is a necessary condition essential to be able to read an EEG. It appeared important for us to be able to clearly identify them using surface electrodes in a context without anesthesia. The physiological rhythms described in the literature (33–36) for stud and cup electrodes were visualized in both species, regardless of conformation, sex, or age. Drowsiness, observed in 84% of dog EEGs and 82% of cat EEGs with cup electrodes, also serves as a marker of good tolerance and wellbeing of the animal during recording.

Routine EEG in human medicine includes the use of two provocative methods for seizure induction: hyperventilation and IPS. Hyperventilation is not possible in animals, as well as in young children, since it requires the patient to voluntarily perform deep breaths. Therefore, we focused on IPS with a protocol of frequency flashes of 3–5–7–10–13–15–17–20–25–30–35–40–45–50 Hz. The IPS protocols suggested in veterinary articles are variable, and no argument favors one over the others (44, 46, 47, 54, 55). Recent recommendations in human medicine suggest performing frequency flashes of 1–2–8–10–15–18–20–25–40–50–60 Hz (6) without us being able to determine if this protocol would be the best for use in pets. It is also indicated that IPS for human should be done with both eyes closed and eyes open. However, it is challenging to request this from animals, and manually closing their eyes can lead to movements that generate artifacts. Nevertheless, we have observed that animals naturally close and open their eyes during IPS. Therefore, we found that IPS was a stimulation technique very well-tolerated and easy to use in awake animals.

In the large proportion of cat EEGs (85% of cases), high-amplitude graphoelements were observed synchronously and with the same frequency as the light flashes, identical in appearance to the photic driving described in some humans. This particularity in cats has previously been described during examinations under anesthesia (47), but its significance is unknown, as are its medical implications in cats that do not show this training. In dogs, this phenomenon has been observed in 11% of cases and has also been previous described (56).

Epileptic discharges were observed during IPS in six dogs, associated with myoclonic responses for five dogs. For two dogs, anomalies were only observed during IPS, which justifies its use in dog EEG.

As recommended (6) and since the animals were not anesthetized, we also conducted stimulations known to provoke seizures as reported by the owner, such as noise or meal stimuli. We observed epileptic discharges in one dog EEG following a meal and in one dog and one cat EEGs following noise emission. Our EEG method without anesthesia enables the confirmation of reflex epilepsies, documented in both dogs (7, 57, 58) and cats (59).

Our population included animals with paroxysmal manifestations compatible with epileptic seizures, episodes of dyskinesia or compulsive disorders, and others without paroxysmal manifestations but with confusional states or vestibular involvement. Within this large population, 31.6% of EEG recordings allowed for the establishment or clarification of diagnoses in different ways: by establishing a correlation between clinical symptoms and electrical anomalies observed on the EEG, by demonstrating the absence of electrical abnormalities during clinical paroxysmal event, or by revealing interictal epileptic discharges. One study reports a diagnostic EEG rate under ambulatory conditions without anesthesia of 68% (43/63) in a population of dogs with a history of paroxysmal events (43). However, comparison of our results is difficult because we included animals with a history of paroxysmal events, some of whom were treated with anti-epileptic drugs, and animals with encephalopathy or vestibular disorders. Some studies report epileptic discharges in up to 50% of healthy dogs under anesthesia (27, 54). In our study, none of the five dogs in the control group exhibited any abnormalities on the EEG. This highlights the need to use standardized protocols, overcoming the variability associated with anesthetic protocols electrode and criteria for interpreting EEG to perform examinations on both healthy and diseased animals. These results have led us to develop the first guidelines for routine EEG recording (Table 3) in an easy, fast and reproducible manner. Thanks to this methodology, routine EEG, performed non-invasively and without anesthesia, could be offered before MRI and cerebrospinal fluid analysis as recommended by an International Veterinary Epilepsy Task Force Consensus report (21). It can enable diagnosis as well as appropriate therapeutic management of cerebral dysfunction and stabilization of the animal's condition, allowing for a comprehensive investigation of any structural causes under better conditions.

**Table 3:** Protocol recommendations.

**Examination facilities**
• Unstimulating and peaceful environment, darkness possible • Fed animal, access to a place of disposal before the examination, sleeping place • Involvement of the owner to avoid disturbances and facilitate the relaxation of the animal
**Course of the recording**
• 45 min minimum • Check impedance < 10 kΩ, < 20 kΩ accepted • IPS if possible • Interventions limited on the animal • Recording of drowsiness patterns required, sleep if possible • Real-time clinical and EEG events reporting
**Equipment**
• 8 gel EEG electrodes • Electrodes headset • 2 EMG • 1 ECG • Breathing belt • Video EEG • Synchronized photic stimulation lamp • Wired or unwired device
**EEG evaluation and interpretation**
• Quality score including impedance, numbers of EEG channels, artifact percentage • Use of a defined and accepted terminology • Description of artifacts, background rhythm, physiological and pathological graphoelements • Electroclinical correlation • Expertise

Our study is a promising step toward the widespread use of EEG in common practice for neurological diagnosis with an easy, non-invasive protocol using surface electrodes and no anesthesia. This protocol is particularly suitable for dogs and cats, for which EEG in clinical practice is not at all developed and would enable electro-clinical characterization of epilepsies.

## Data availability statement

The raw data supporting the conclusions of this article will be made available by the authors, without undue reservation.

## Ethics statement

The animal studies were approved by the Ethics Committee of VetAgro Sup (No. 18). The studies were conducted in accordance with the local legislation and institutional requirements. Written informed consent was obtained from the owners for the participation of their animals in this study.

## Author contributions

EL: Conceptualization, Data curation, Formal analysis, Investigation, Methodology, Visualization, Writing—original draft, Writing—review & editing. HP: Funding acquisition, Project administration, Resources, Supervision, Writing—review & editing. SBl: Investigation, Methodology, Writing—review & editing. TT: Investigation, Methodology, Writing—review & editing. NV: Investigation, Methodology, Writing—review & editing. SBe: Conceptualization, Formal analysis, Methodology, Supervision, Validation, Writing—review & editing. CE: Conceptualization, Formal analysis, Investigation, Methodology, Supervision, Validation, Writing—review & editing.
